# Preventive and Therapeutic Efficacy of Finasteride and Dutasteride in TRAMP Mice

**DOI:** 10.1371/journal.pone.0077738

**Published:** 2013-10-18

**Authors:** Alexander B. Opoku-Acheampong, Dave Unis, Jamie N. Henningson, Amanda P. Beck, Brian L. Lindshield

**Affiliations:** 1 Department of Human Nutrition, Kansas State University, Manhattan, Kansas, United States of America; 2 Division of Biology, Kansas State University, Manhattan, Kansas, United States of America; 3 College of Veterinary Medicine, Kansas State University, Manhattan, Kansas, United States of America; 4 Section of Comparative Medicine, Yale University School of Medicine, New Haven, Connecticut, United States of America; University of L'Aquila, Italy

## Abstract

**Background:**

The prostate cancer prevention trial (PCPT) and Reduction by dutasteride of Prostate Cancer Events (REDUCE) trial found that 5α-reductase (5αR) inhibitors finasteride and dutasteride respectively, decreased prostate cancer prevalence but also increased the incidence of high-grade tumors. 5αR2 is the main isoenzyme in normal prostate tissue; however, most prostate tumors have high 5αR1 and low 5αR2 expression. Because finasteride inhibits only 5αR2, we hypothesized that it would not be as efficacious in preventing prostate cancer development and/or progression in C57BL/6 TRAMP x FVB mice as dutasteride, which inhibits both 5αR1 and 5αR2.

**Method/Principal Findings:**

Six-week-old C57BL/6 TRAMP x FVB male mice were randomized to AIN93G control or pre- and post- finasteride and dutasteride diet (83.3 mg drug/kg diet) groups (n =30–33) that began at 6 and 12 weeks of age, respectively, and were terminated at 20 weeks of age. The pre- and post- finasteride and dutasteride groups were designed to test the preventive and therapeutic efficacy of the drugs, respectively. Final body weights, genitourinary tract weights, and genitourinary tract weights as percentage of body weights were significantly decreased in the Pre- and Post-dutasteride groups compared with the control. The Post-dutasteride group showed the greatest inhibition of prostatic intraepithelial neoplasia progression and prostate cancer development. Surprisingly, the Post-dutasteride group showed improved outcomes compared with the Pre-dutasteride group, which had increased incidence of high-grade carcinoma as the most common and most severe lesions in a majority of prostate lobes. Consistent with our hypothesis, we found little benefit from the finasteride diets, and they increased the incidence of high-grade carcinoma.

**Conclusion:**

Our findings have commonalities with previously reported PCPT, REDUCE, and the Reduction by dutasteride of Clinical Progression Events in Expectant Management (REDEEM) trial results. Our results may support the therapeutic use of dutasteride, but not finasteride, for therapeutic or preventive use.

## Introduction

Prostate cancer is the most commonly diagnosed non-skin neoplasm in men and is projected to account for 28% of US male cancer cases in 2013 [1]. Most prostate tumor growth is initially androgen-dependent or androgen-sensitive [2]. The main circulating androgen, testosterone, is converted to dihydrotestosterone by the isoenzymes 5α-reductase 1 and 5α-reductase 2. Dihydrotestosterone has up to a ten-fold higher affinity to the androgen receptor than testosterone, making it a more potent androgen [3,4]. 5α-reductase 2 is the major isoenzyme in the prostate [5]; however, multiple [6-9], but not all [10-12], studies have reported increased 5α-reductase 1 and/or decreased 5α-reductase 2 mRNA expression or activity in prostate cancer compared with nonmalignant prostate tissue. Furthermore, 5α-reductase 1 and 5α-reductase 2 were found in 73% and 56%, respectively, of human prostate cancer tissues [11].

 Finasteride (5α-reductase 2 inhibitor) and dutasteride (5α-reductase 1 and 2 inhibitor) are commonly used to treat benign prostatic hyperplasia (BPH), a nonmalignant enlargement of the prostate. The potential of these inhibitors to decrease prostate cancer development and/or progression through their anti-androgen action has been examined in several clinical trials. The Prostate Cancer Prevention Trial (PCPT) and the Reduction by Dutasteride of Prostate Cancer Events (REDUCE) trial found that finasteride and dutasteride decreased prostate cancer risk by 24.8% and 23%, respectively, but both inhibitors also increased the risk of developing high-grade prostate cancer [13,14]. As a result, the Food and Drug Administration (FDA) amended the safety information for both drugs to state that they increase high-grade prostate cancer in patients [15]. In addition, it has been projected that finasteride and dutasteride in PCPT and REDUCE trials respectively showed no prostate cancer mortality benefit [16]. Another clinical trial, the Reduction by Dutasteride of Clinical Progression Events in Expectant Management (REDEEM) trial found that dutasteride significantly delayed prostate cancer progression with no reported adverse events in men with low-risk, localized prostate cancer [17].

 In animal models, dutasteride, but not finasteride, decreased Dunning R-3327H rat prostate tumor weights [18]. Similarly, Canene-Adams and colleagues also reported that finasteride did not alter Dunning R-3327H rat prostate tumor areas or weights despite reducing androgen-sensitive tissue weights [19]. Finasteride also did not decrease prostatic intraepithelial neoplasia (PIN) or adenocarcinoma in 10-week-old transgenic rats bearing the probasin/simian virus 40 T antigen (SV40 Tag) construct but did decrease lesion size in lateral and ventral lobes, but not the dorsal lobe, of the prostate [20]. Both finasteride and dutasteride were effective in reducing LNCaP human prostate cancer xenograft growth in male nude mice [18]. Dutasteride significantly decreased LuCaP 35 tumor growth in Balb/c mice [21]. Previously, we examined the effects of finasteride and dutasteride diets begun 1-2 weeks before or 3 weeks after subcutaneous injection of WPE1-NA22 human prostate cancer cells in male nude mice, but we were unable to answer our research question due to poor tumor growth [22]. 

 Thus, we decided to determine the effects of finasteride and dutasteride in transgenic adenocarcinoma of the mouse prostate (TRAMP) mice since prostate cancer development and progression have been well characterized in this model [23]. TRAMP mice prostate cancer is promoted by the expression of the SV40 large and small T antigen and undergoes progressive stages of cancer development starting from prostatic intraepithelial neoplasia (PIN) to adenocarcinoma and metastasis [24,25]. In this study, we compared the effects of finasteride- or dutasteride-containing diets begun at 6 weeks or 12 weeks of age on prostate tumor development in C57BL/6 TRAMP x FVB mice. These time points were chosen because 6 weeks is when the mice reach sexual maturity and develop pathologic features similar to low-grade PIN [26]. This would allow us to determine whether finasteride and/or dutasteride can inhibit PIN progression and prostate cancer development. The post- finasteride and dutasteride diets began at 12 weeks of age when mice are expected to have developed PIN and well-differentiated prostate cancer [25]. Beginning diets at this age would allow us to determine whether therapeutic finasteride and/or dutasteride can inhibit PIN and/or prostate cancer progression. Because of the increase in 5α-reductase 1 and decrease in 5α-reductase 2 activity and expression that may occur during prostate cancer development, we hypothesized that finasteride diets begun at either time point would not and dutasteride diets begun at either time point would significantly inhibit prostate cancer development and/or progression.

## Materials and Methods

### Ethics Statement

The Institutional Animal Care and Use Committee (IACUC) at Kansas State University approved all animal procedures (protocol 2969).

### Study Mice, Diets, and Design

**Figure 1:**
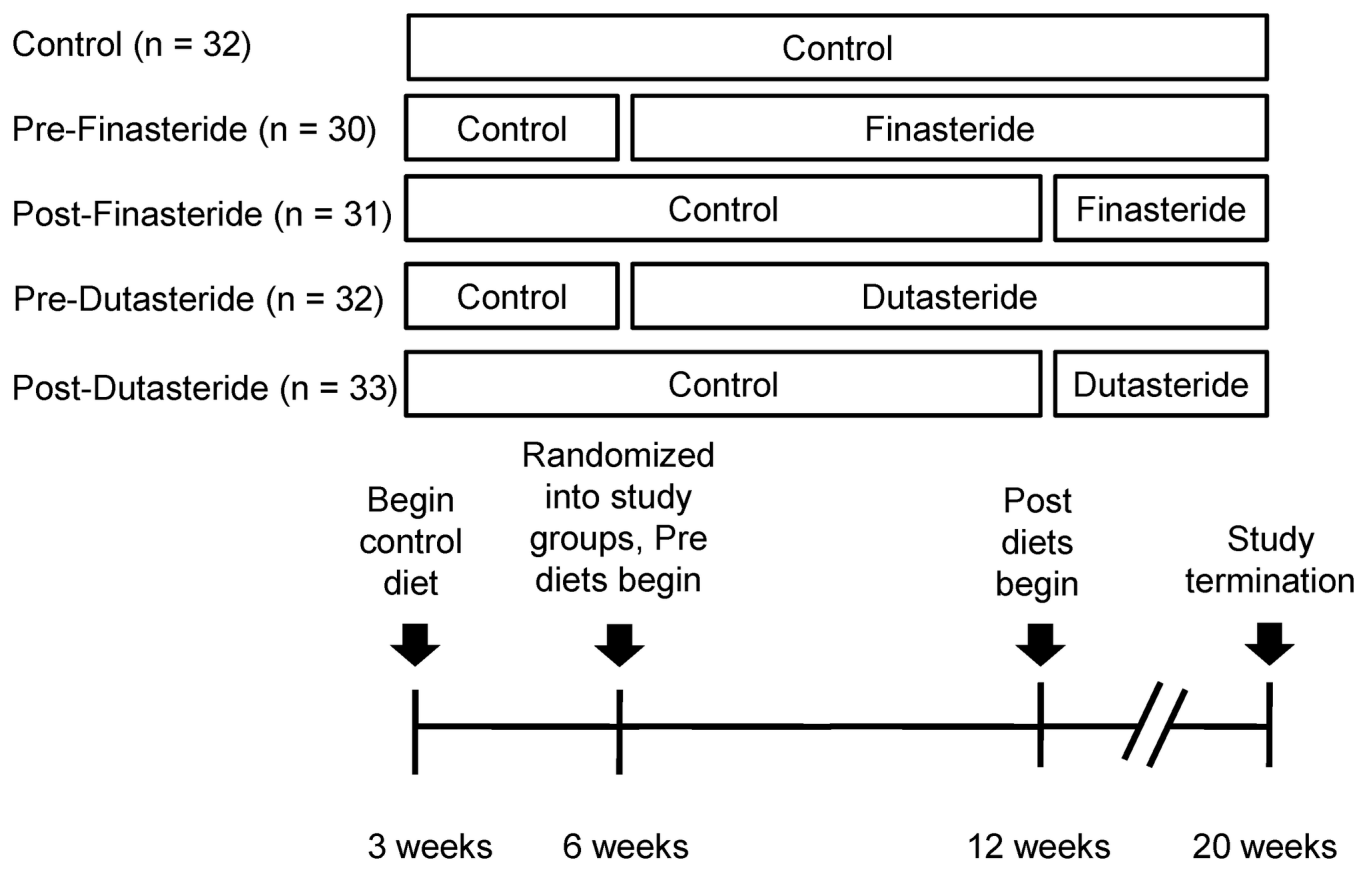
Study design Male C57BL/6 TRAMP x FVB mice were weaned at 3 weeks of age, fed a control diet, and randomized into Control, Pre-Finasteride, Post-Finasteride, Pre-Dutasteride, and Post-Dutasteride groups (n = 30–33) at 6 weeks of age. Pre- and post-groups began their treatment diets at 6 and 12 weeks, respectively, and the study was terminated when mice were 20 weeks of age.

Six-week-old heterozygous C57BL/6-Tg 8247Ng/J TRAMP male and female mice were purchased (The Jackson Laboratory, Bar Harbor, ME) and bred to produce homozygous males. These were bred with female FVB/NJ mice (The Jackson Laboratory, Bar Harbor, ME) to produce C57BL/6 TRAMP x FVB mice. Male C57BL/6 TRAMP x FVB mice were weaned and began consuming the control diet at 3 weeks of age before being randomized into Control, Pre-Finasteride, Post-Finasteride, Pre-Dutasteride, and Post-Dutasteride groups (n = 30–33) at 6 weeks of age. Mice were individually housed, monitored daily, weighed weekly, and provided diets and water *ad libitum*. AIN93-G treatment diets (Research Diets, New Brunswick, NJ) contained dutasteride (kindly donated by GlaxoSmithKline Pharmaceuticals, Research Triangle Park, NC) and finasteride (Kemprotec, Middlesbrough, UK) at 83.3 mg/kg of diet, the same dose used in our previous study [22]. These diets were designed to provide ~10mg drug/kg body weight, which was the midrange dutasteride dose provided by Xu and colleagues [18]. Pre- and post-groups began their treatment diets at 6 weeks and 12 weeks of age, respectively (Figure 1). Seven mice did not complete the study for health reasons unrelated to tumor growth, leaving the group numbers shown in Figure 1. At 20 weeks of age, mice were anesthetized by CO_2_ inhalation and euthanized by exsanguination. The genitourinary tracts, kidneys, and lungs were dissected, and the genitourinary tracts were weighed. Iliac lymph nodes were also collected whenever possible. All tissues were fixed by immersion in 10% neutral buffered formalin for 48 hours, and then moved to 70% alcohol until processing in the Kansas State University Veterinary Diagnostic Laboratory.

### Histopathology

The seminal vesicles; anterior, dorsal, lateral, and ventral prostate lobes; ampulla; urinary bladder; and proximal urethra were sampled as one tissue. Orientation was maintained, and the dorsal side was placed down in the cassette for histology processing first. In mice with prostate tumors, distinct prostate lobes were not recognizable, so a section was taken through the center of the mass. Tissues were routinely processed and embedded in paraffin wax. Sections were made at 4 µm, routinely processed, and stained with hematoxylin and eosin. After the dorsal sections were processed, the blocks were melted down and the tissue was flipped and re-embedded for examination of the ventral prostate. Prostate lesions were scored blindly twice by a board-certified veterinary pathologist (JNH) according to a previously described grading scheme [27]. In short, both the most common and most severe lesions were graded separately, and then adjusted for distribution. If a neoplasm replaced all four prostate lobes, then all lobes received the same score. Select tissues were also blindly scored twice by a second board-certified veterinary pathologist (APB) to ensure scoring consistency. Iliac lymph nodes were examined for metastasis by removal of the kidneys and sublumbar tissue; histological processing was identical to the prostatic tissue. Photomicrographs were taken with an Olympus DP26 digital camera (Olympus America, Center Valley, PA) with a 40X objective, giving 0.75µm resolution.  Images were captured with Olympus cellSens software.

### Statistical Analysis

Data were analyzed using SAS 9.3 (SAS Institute Inc., Cary, NC) with p<0.05 considered statistically significant. Natural logs were used to transform data that did not meet model assumptions. Data were analyzed using ANOVA with Fisher’s Least Significant Difference (LSD). Iliac lymph node metastases incidence was analyzed using the Kruskal Wallis non-parametric one-way ANOVA.

## Results

### Final body weights and genitourinary tract weights

**Figure 2:**
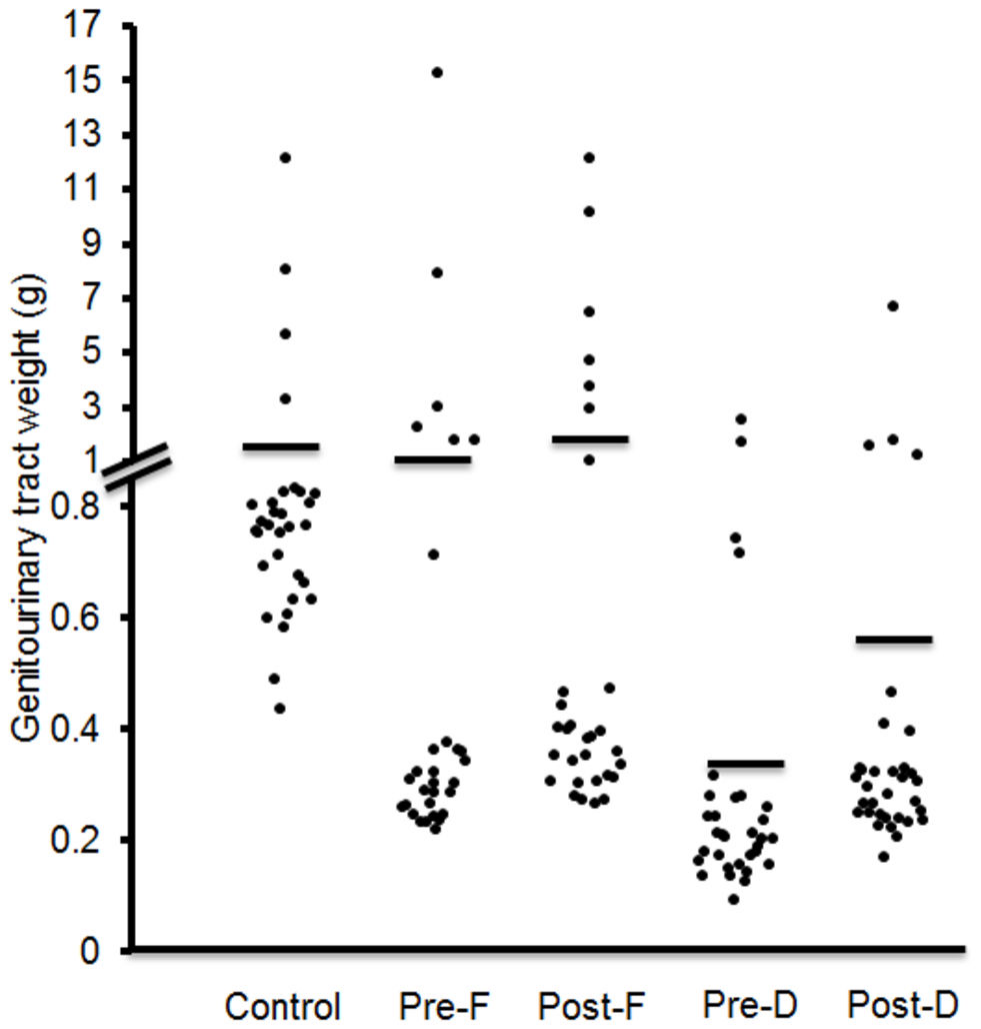
Genitourinary tract weights. Solid lines indicate mean values (n = 30–33). Pre-F = Pre-Finasteride, Post-F = Post-Finasteride, Pre-D = Pre-Dutasteride, Post-D = Post-Dutasteride.

Pre- and Post-Dutasteride groups’ final body weights were significantly decreased compared with the control despite no significant difference in daily food intake (Table 1); however, both dutasteride diets significantly decreased the weight gain/food intake ratio versus the control and Pre-Finasteride group. While it is a small numerical difference, the Pre-Finasteride group’s daily food intake was significantly higher than the control and both dutasteride groups. Genitourinary tract weights for Pre- and Post-Dutasteride groups also were significantly lower than the control (Table 1 and Figure 2). Genitourinary tract weights as percentage of body weights in the Pre- and Post-Dutasteride groups were also significantly lower than the control and Post-Finasteride group; thus the significant decrease in genitourinary tract weights was not due to the decreased body weights in these groups. Both dutasteride groups’ genitourinary tract weights were significantly decreased compared with the Post-Finasteride group; the Pre-Dutasteride group’s genitourinary tract weights and genitourinary tract weights as percentage of body weights were also significantly decreased compared with the Pre-Finasteride group. The Pre-Finasteride group’s genitourinary tract weights as percentage of body weights also were significantly decreased compared with the control.

**Table 1:** Final body weights, daily food intake, weight gain/food intake ratio, genitourinary tract weights, and genitourinary tract weights as percentage of body weights (n = 28-33)^1^

**Group**	**Final body weights (g)**	**Daily food intake (g)**	**Weight gain/food intake ratio (g gained/g total food intake x 100)**	**Genitourinary tract weights (g)**	**Genitourinary tract weights as percentage of body weights**
Control	33.3 ± 0.5^a^	2.99 ± 0.03^a^	2.8 ± 0.2^a^	1.56 ± 0.46^a^	4.91 ± 1.51^a^
Pre-Finasteride	33.7 ± 0.6^a^	3.08 ± 0.02^b^	2.8 ± 0.2^a^	1.31 ± 0.55^a,b^	3.50 ± 1.32^b,d^
Post-Finasteride	33.0 ± 0.6^a,b^	3.02 ± 0.04^a,b^	2.7 ± 0.2^a^,^b^	1.65 ± 0.55^a^	4.87 ± 1.55^a,b^
Pre-Dutasteride	29.9 ± 0.4^c^	2.96 ± 0.02^a^	1.6 ± 0.1^c^	0.35 ± 0.09^c^	1.19 ± 0.31^c^
Post-Dutasteride	31.8 ± 0.5^b^	2.96 ± 0.02^a^	2.4 ± 0.1^b^	0.59 ± 0.20^b,c^	1.88 ± 0.63^c,d^

^1^ Data are mean ± SEM; values with different letters are statistically different (p<0.05).

### Most severe lesion scores

**Figure 3:**
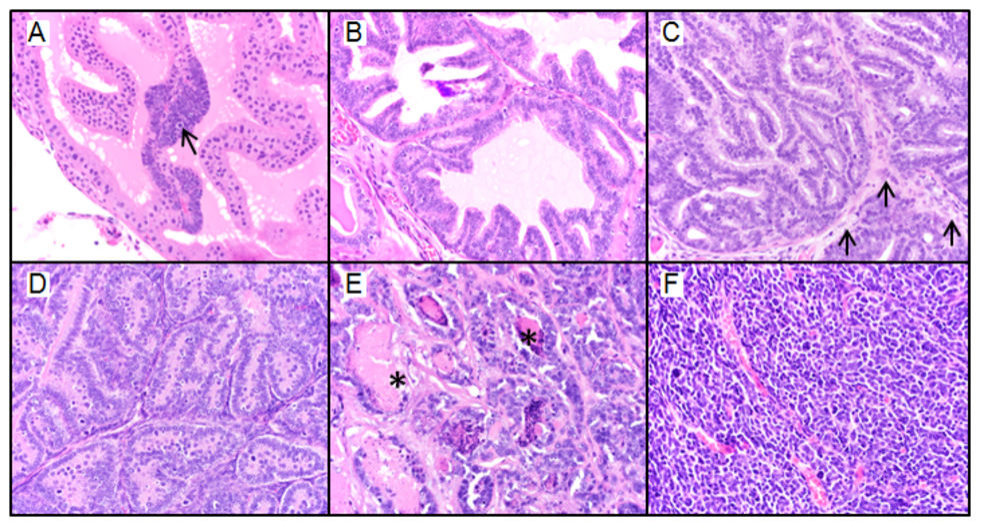
Prostate pathology in 20-week-old C57BL/6 TRAMP x FVB male mice captured at 40X magnification. (A) Grade 1, low-grade PIN. There is focal hyperplasia of prostate epithelial cells resulting in stratification of cells (arrow). Hyperplastic cells have increased basophilia and increased nuclear to cytoplasmic ratios. (B) Grade 2, moderate-grade PIN. Hyperplastic epithelial cells form increased numbers of short and tall papillary projections that extend into the glandular lumen. (C) Grade 3, high-grade PIN. There is loss of prostate glandular lumina due to the presence of numerous hyperplastic prostate epithelial cells that project into the lumen and form a cribiform pattern. Hyperplastic cells do not invade the connective tissue that separates the glands into distinct lobules (arrows). (D) Grade 5, Well-differentiated adenocarcinoma. Well-differentiated neoplastic cells form tubular or glandular like structures that have obliterated lobular architecture by invasion of the connective tissue borders; lobules cannot be observed in this photomicrograph as compared to photomicrograph C. Necrosis of neoplastic cells is absent. (E) Grade 6, Moderately-differentiated adenocarcinoma. Neoplastic prostate epithelial cells are attempting to form glandular structures. Glandular structures vary in size and shape. Cellular atypia is increased and necrosis is present (asterisks). (F) Grade 7, Poorly-differentiated carcinoma. Neoplastic cells have marked atypia and are arranged in sheets with no attempt at forming glandular or tubular structures as compared to figures D and E.

The raw and adjusted mean most severe lesion scores for the anterior, dorsal, and lateral lobes were significantly decreased in the Post-Dutasteride group versus the control (Table 2). Representative images of the different pathological grades used in the grading scheme are shown in Figure 3. The adjusted mean most severe lesion score was also significantly decreased for the ventral lobe of the Post-Dutasteride group compared with the control. The raw and adjusted mean most severe lesion scores for the anterior and dorsal lobes of the Post-Dutasteride groups were also significantly decreased compared with the finasteride groups. The raw and adjusted mean most severe lesion scores for the anterior and dorsal lobes of the Pre-Dutasteride group were significantly decreased versus the control. The lateral lobe adjusted mean most severe lesion score was also significantly decreased in the Pre-Dutasteride group compared with the control. There were also no significant differences in raw and adjusted mean most severe lesion scores between the finasteride groups and the control.

**Table 2:** Raw and adjusted mean most severe lesion scores for the anterior, dorsal, lateral, and ventral prostate lobes (n = 28–33) ^1^.

	**Anterior prostate**		**Dorsal prostate**		**Lateral prostate**		**Ventral prostate**	
**Group**	**Raw**	**Adjusted**	**Raw**	**Adjusted**	**Raw**	**Adjusted**	**Raw**	**Adjusted**
Control	3.36 ± 0.30^a^	8.69 ± 0.99^a^	3.56 ± 0.28^a^	9.52 ± 0.86^a^	3.45 ± 0.34^a^	9.45 ± 1.05^a^	3.03 ± 0.36	8.05 ± 1.13^a^
Pre-Finasteride	3.20 ± 0.38^a^	8.92 ± 1.26^a^	3.31 ± 0.44^a,b^	8.74 ± 1.43^a,b^	3.37 ± 0.45^a,b^	9.05 ± 1.46^a,b^	3.53 ± 0.49	9.55 ± 1.58^a,b^
Post-Finasteride	3.03 ± 0.36^a^	7.69 ± 1.15^a^	3.47 ± 0.37^a,b^	9.24 ± 1.20^a^	3.32 ± 0.46^a,b^	8.98 ± 1.46^a,b^	3.26 ± 0.42	8.73 ± 1.35^a,c^
Pre-Dutasteride	2.09 ± 0.30^b^	4.84 ± 0.95^b^	3.06 ± 0.45^b,c^	8.03 ± 1.4^b,c^	3.25 ± 0.47^a,b^	8.52 ± 1.48^b^	3.11 ± 0.47	8.05 ± 1.54^a,b^
Post-Dutasteride	2.21 ± 0.26^b^	5.09 ± 0.82^b^	2.23 ± 0.33^c^	5.53 ± 1.04^c^	2.86 ± 0.43^b^	7.35 ± 1.34^b^	2.53 ± 0.40	6.27 ± 1.28^b^

1 Data are mean ± SEM; values with different letters are statistically different (p<0.05).

### Most common lesion scores

The raw mean most common lesion scores for the anterior and dorsal lobes were significantly decreased in both dutasteride groups versus the control (Table 3). In addition, for all lobes in the Post-Dutasteride group, the adjusted most common lesion scores were significantly decreased compared with the control. The adjusted most common lesion scores for the anterior, dorsal, and lateral lobes in the Pre-Dutasteride group were significantly decreased versus the control. The Post-Dutasteride group anterior lobe raw and adjusted mean most common lesion scores and both dutasteride groups’ dorsal lobe adjusted most common lesion scores were significantly decreased compared with the Post-Finasteride group. The adjusted most common lesion score for the dorsal lobe in the Pre-Finasteride group was significantly decreased versus the control, despite being numerically higher. Transforming the data so that it would meet assumptions resulted in a transformed adjusted mean score that was significantly lower than the control because the transformed score was not as impacted by the high scores in this group.

**Table 3:** Raw and adjusted mean most common lesion scores for the anterior, dorsal, lateral, and ventral prostate lobes (n = 28–33) ^1^.

	**Anterior prostate**		**Dorsal prostate**		**Lateral prostate**		**Ventral prostate**		
**Group**	**Raw**	**Adjusted**	**Raw**	**Adjusted**	**Raw**	**Adjusted**	**Raw**	**Adjusted**
Control	2.55 ± 0.36^a^	6.75 ± 1.15^a^	2.63 ± 0.23^a^	7.41 ± 0.69^a^	2.62 ± 0.32	7.42 ± 1.01^a^	2.72 ± 0.37	7.52 ± 1.19^a^	
Pre-Finasteride	2.38 ± 0.41^a,b^	6.23 ± 1.29^a,b^	2.74 ± 0.47^a,b^	7.43 ± 1.49^b,c^	2.82 ± 0.47	7.70 ± 1.50^a,b^	3.16 ± 0.50	8.64 ± 1.62^a,b^	
Post-Finasteride	2.33 ± 0.38^a,c^	6.00 ± 1.19^a,c^	2.82 ± 0.42^a,b^	7.53 ± 1.26^a,b^	2.88 ± 0.47	7.95 ± 1.48^a,b^	2.95 ± 0.44	8.11 ± 1.41^a^	
Pre-Dutasteride	1.63 ± 0.27^b,c^	3.72 ± 0.85^b,c^	2.32 ± 0.43^b^	6.06 ± 1.37^c^	2.69 ± 0.46	7.02 ± 1.44^b^	2.73 ± 0.46	7.06 ± 1.48^a,b^	
Post-Dutasteride	1.59 ± 0.27^b^	3.68 ± 0.83^b^	2.03 ± 0.33^b^	5.08 ± 1.06^c^	2.33 ± 0.40	5.95 ± 1.27^b^	2.28 ± 0.41	5.72 ± 1.30^b^	

1 Data are mean ± SEM; values with different letters are statistically different (p<0.05).

### Most severe lesion histopathological distribution

 Low-grade (LG) and high-grade (HG) PIN incidence as the most severe lesion increased and decreased, respectively, significantly in all lobes in both dutasteride groups versus the control (Table 4). Low-grade PIN incidence was increased in the ventral lobe of both finasteride groups compared with the control. Lateral and ventral lobe medium-grade (MG)-PIN incidence was also significantly decreased in both dutasteride groups versus the control. Compared with the control, there was a significant increase in MG-PIN incidence in the dorsal lobe of the Pre-Finasteride group. Well-differentiated (WD) adenocarcinoma incidence in the anterior lobe was significantly decreased in both finasteride and dutasteride groups compared with the control, and the Pre-Finasteride group had significantly increased moderately differentiated (MD) adenocarcinoma incidence versus the control in the ventral lobe. These differences are, however, based on low incidence levels. The finasteride groups and the Pre-Dutasteride group had significantly increased poorly differentiated (PD) carcinoma and prostate cancer (WD-PD) incidence in the lateral and ventral lobes versus the control. The finasteride groups and the Pre-Dutasteride group also had increased poorly differentiated (PD) carcinoma in the dorsal lobe versus the control. The Pre-Finasteride and Pre-Dutasteride groups had increased prostate cancer (WD-PD) incidence in the dorsal lobe versus the control. The Post-Dutasteride group had significantly decreased and increased PD carcinoma incidence in the anterior and lateral lobes, respectively, compared with the control. Both dutasteride groups had significantly decreased prostate cancer (WD-PD) incidence in the anterior lobe compared with the control. 

**Table 4:** Histopathological analysis (most severe lesion) of individual prostate lobes in control, finasteride, and dutasteride groups ^1, 2^.

			**PIN**				**Adenocarcinoma**				**Prostate cancer**	
	**n**			**LG**	**MG**	**HG**		**WD**	**MD**	**PD**		**(WD-PD)**
**Anterior prostate**												
Control		32		2%^a^	30%	50%^a^		3% ^a^	0%	16%^a,b^		19%^a^
Pre-Finasteride		30		15%^a,b^	32%	31%^a,b^		0%^b^	2%	20%^a^		22%^a^
Post-Finasteride		29		14%^a^	40%	28%^a,b^		0%^b^	0%	17%^a^		17%^a^
Pre-Dutasteride		32		49%^c^	35%	5%^b^		0%^b^	0%	11%^b,c^		11%^b^
Post-Dutasteride		33		33%^b,c^	48%	9%^b^		0%^b^	0%	9%^c^		9%^b^
**Dorsal prostate**												
Control		32		2%^a^	11%^a^	69%^a^		2%	2%	16%^a^		19%^a^,^c^
Pre-Finasteride		29		17%^a^	45%^b^	10%^b,c^		0%	0%	28%^b^		28%^b^
Post-Finasteride		31		5%^a^	40%^a,b^	31%^b^		0%	0%	24%^b^		24%^b^,^c^
Pre-Dutasteride		31		42%^b^	26%^a,b^	3%^c^		0%	0%	29%^b^		29%^b^
Post-Dutasteride		33		43%^b^	38%^a,b^	6%^c^		0%	0%	12%^a^		12%^a^
**Lateral prostate**												
Control		28		11%^a^	46%^a^	23%^a^		0%	0%	20%^a^		20%^a^
Pre-Finasteride		30		23%^a^	43%^a^	2%^b^		0%	0%	32%^b^		32%^b^
Post-Finasteride		30		27%^a,b^	35%^a,b^	8%^b^		0%	0%	30%^b,c^		30%^b^,^c^
Pre-Dutasteride		32		42%^b,c^	22%^b^	3%^b^		0%	0%	33%^b^		33%^b^
Post-Dutasteride		33		48%^c^	20%^b^	6%^b^		0%	0%	26%^c^		26%^c^
**Ventral prostate**												
Control		30		0%^a^	35%^a^	45%^a^		0%	0%^a^	20%^a^		20%^a^
Pre-Finasteride		28		29%^b^	24%^a,b,c^	9%^b^		0%	4%^b^	35%^b^		38%^b^
Post-Finasteride		30		22%^b^	31%^a,b^	22%^c^		0%	0%^a^	25%^c^		25%^c^
Pre-Dutasteride		31		46%^c^	21%^b,c^	0%^d^		2%	0%^a^	31%^b^		33%^d^
Post-Dutasteride		30		51%^c^	13%^c^	15%^b,c^		0%	0%^a^	20%^a^		20%^a^

1 Values with different letters are statistically different from (p<0.05).

2 LG = low-grade, MG = moderate-grade, HG = high-grade, PIN = prostatic intraepithelial neoplasia, WD = well-differentiated,

MD = moderately differentiated, PD = poorly differentiated.

### Most common lesion histopathological distribution

 As was observed in the most severe lesion scores, LG-PIN as the most common lesion was significantly increased in all lobes in both dutasteride groups versus the control (Table 5). Both finasteride groups also had significantly increased LG-PIN incidence in the dorsal, lateral, and ventral lobes compared with the control. MG-PIN incidence was also significantly decreased for the Pre-Finasteride group and both dutasteride groups in the dorsal, ventral, and lateral lobes versus the control. The Post-Finasteride group had significantly decreased MG-PIN incidence in the ventral and lateral lobes compared with the control. Both dutasteride groups had significantly decreased incidence of HG-PIN versus the control in the anterior, dorsal and lateral lobes. Both finasteride groups had significantly decreased HG-PIN compared with the control in the dorsal and lateral lobes. Both dutasteride groups had significantly decreased incidence of PD carcinoma in the anterior lobe versus the control. Both finasteride groups and the Pre-Dutasteride group had significantly increased PD carcinoma incidence compared with the control in the dorsal, lateral, and ventral lobes. Compared with the control, a significant increase in PD carcinoma incidence was observed in the lateral prostate of the Post-Dutasteride group. Total prostate cancer (WD-PD) was almost entirely composed of PD carcinoma, so the significant differences compared with the control were the same as those seen in PD carcinoma for the anterior, dorsal and lateral lobes.

**Table 5:** Histopathological analysis (most common lesion) of individual prostate lobes in control, finasteride, and dutasteride groups ^1, 2^.

			**PIN**				**Adenocarcinoma**				**Prostate cancer**	
	**n**			**LG**	**MG**	**HG**		**WD**	**MD**	**PD**		**(WD-PD)**
**Anterior prostate**												
Control		32		44%^a^	20%	20%^a^		0%	0%	16%^a^		16%^a^
Pre-Finasteride		30		56%^a^	22%	3%^a^,^b^		0%	0%	19%^a^		19%^a^
Post-Finasteride		29		50%^a^	29%	5%^a^,^b^		0%	0%	16%^a^		16%^a^
Pre-Dutasteride		32		76%^b^	16%	0%^b^		0%	0%	8%^b^		8%^b^
Post-Dutasteride		33		79%^b^	14%	0%^b^		0%	0%	8%^b^		8%^b^
**Dorsal prostate**												
Control		32		5%^a^	59%^a^	28%^a^		0%	0%	8%^a^		8%^a^
Pre-Finasteride		29		55%^b^	19%^b,c^	0%^b^		0%	0%	26%^b^		26%^b^
Post-Finasteride		31		33%^c^	44%^a,d^	2%^b^		0%	0%	21%^b^		21%^b^
Pre-Dutasteride		31		73%^b^	6%^c^	0%^b^		0%	0%	21%^b^		21%^b^
Post-Dutasteride		33		57%^b^	31%^b,d^	0%^b^		0%	0%	12%^a^		12%^a^
**Lateral prostate**												
Control		28		10%^a^	70%^a^	7%^a^		0%	0%	14%^a^		14%^a^
Pre-Finasteride		30		52%^b^	22%^b^	0%^b^		0%	0%	27%^b^		27%^b^
Post-Finasteride		30		47%^b^	25%^b^	2%^b^		0%	0%	27%^b^		27%^b^
Pre-Dutasteride		32		64%^c^	9%^c^	0%^b^		0%	0%	27%^b^		27%^b^
Post-Dutasteride		33		65%^c^	15%^b,c^	0%^b^		0%	0%	20%^c^		20%^c^
**Ventral prostate**												
Control		30		14%^a^	68%^a^	2%		0%	0%	16%^a^		16%^a^
Pre-Finasteride		28		45%^b^	20%^b^	0%		0%	0%	35%^b^		35%^b^
Post-Finasteride		30		32%^c^	43%^c^	0%		0%	0%	25%^c^		25%^c^,^d^
Pre-Dutasteride		31		69%^d^	2%^d^	0%		2%	0%	28%^c^		30%^b^,^d^
Post-Dutasteride		30		64%^d^	17%^b^	0%		0%	0%	19%^a^		19%^a,c^

1 Values with different subscript letters are statistically different from one another (p<0.05).

2 LG = low-grade, MG = moderate-grade, HG = high-grade, PIN = prostatic intraepithelial neoplasia, WD = well-differentiated,

MD = moderately differentiated, PD = poorly differentiated.

### Iliac lymph node metastases

 No significant differences were observed in the incidence of iliac lymph node metastases between groups (Table 6); however, a notable difference was found in incidence between Post-Dutasteride (8%) and Post-Finasteride (29%) groups.

**Table 6:** Iliac lymph node metastases incidence in control, finasteride, and dutasteride groups.

**Group**	**n**	**Lymph node metastases incidence (%)**	
Control	21		4 (19)
Pre-Finasteride	26		5 (19)
Post-Finasteride	24		7 (29)
Pre-Dutasteride	22		5 (23)
Post-Dutasteride	24		2 (8)

## Discussion

Although the efficacy of finasteride and dutasteride in inhibiting tumor growth has been compared, we believe we are the first to determine the effectiveness of finasteride and dutasteride on PIN progression and prostate cancer development in C57BL/6 TRAMP x FVB mice. Overall, we found that the Post-Dutasteride group had the greatest decrease in PIN progression and prostate cancer development. Although not significant, we observed the lowest level of lymph node metastases in this group. We unexpectedly found that the Post-Dutasteride group had better outcomes than the Pre-Dutasteride group, primarily as a result of the higher incidence of PD carcinoma in most lobes in the Pre-Dutasteride group. Both finasteride groups had decreased incidence of HG-PIN, but not to the extent seen in the dutasteride groups. The finasteride groups also had increased incidence of poorly differentiated prostate cancer in most lobes. 

Our findings are similar to results in other animal models that found finasteride failed to inhibit prostate cancer progression [20] or tumor growth [18,19] and dutasteride decreased prostate tumor growth [18]. We found the weakest response to dutasteride in the ventral lobe. Another study that used the same scoring system also found fewer statistical differences in the ventral lobe in response to tomato powder and soy germ diets, especially compared with the dorsal and lateral lobes [28]. This decrease in efficacy in the ventral lobe might be explained by the fact that in large probasin-large T antigen mice, dutasteride markedly decreased the dorsolateral prostate weights but had little to no effect on ventral prostate weights [29]. In addition, rat ventral prostate has almost two-fold higher concentrations of testosterone and dihydrotestosterone than the dorsolateral prostate; furthermore, after finasteride administration, ventral prostate lobe concentrations of testosterone are almost twice as high as dorsolateral prostate lobe concentrations [30]. Another possible explanation for reduced sensitivity in the ventral prostate lobes is that the transgene is expressed at much higher levels in the ventral lobe [31]. 

We were surprised that both dutasteride groups had decreased body weights and weight gain/food intake ratios compared with the control. In our previous study, this diet had no effect on body weights or weight gain/food intake ratios in nude mice despite a longer feeding duration [22]. However, dutasteride has decreased body weight or body weight gain in other studies. For example, dutasteride administration at doses of 2.5 and 5 mg/kg body weight to 8-week-old male Sprague–Dawley rats for 2 weeks led to a significant decrease in their body weights [32], and 4 or 8 weeks of infusion of 2 mg/kg body weight dutasteride treatment led to a significant decrease in body weight gain in large probasin-large T antigen transgenic mice [29]. In our study, the mice were consuming ~8 mg/kg finasteride and dutasteride/day. This is obviously higher than the concentration in previous studies. We did not find an increase in body weights in the Pre-Finasteride group, unlike in our previous study; however, there was a significant increase in food intake versus the control. The lack of an increase in body weights in the Pre-Finasteride group in this study supports our belief that the significant increase in body weights in our previous study was not due to the treatment diet [22].

In our previous study, both finasteride and dutasteride decreased prostate and seminal vesicle weights as percentage of body weights. Dutasteride also significantly decreased seminal vesicle weights compared to finasteride [22]. In the current study, we think prostate tumor development is the reason we did not find significant decreases in genitourinary tract weights in the finasteride groups. The rates of lymph node metastases found in this study are similar to those that have been reported in 18-week-old [28] and 18–24-week-old C57BL/6 TRAMP x FVB mice [33]. 

We are one of the first to use a new grading scheme for TRAMP mice [27]. When comparing our results to those reported previously, the most notable difference is that we had a lower incidence of PD carcinoma as the most severe lesion compared with 18-week-old C57BL/6 TRAMP x FVB mice [28] and most severe and most common lesions in 18–24-week-old C57BL/6 TRAMP x FVB mice [27]. Instead, we had much higher incidence of PIN as the most severe and most common lesions. Our average most severe lesion scores were also lower than those for 10-week-old C57BL/6 TRAMP x FVB mice, but the average most common lesion scores were similar [27]. It is not clear why our scores were lower using this grading scheme, but variation in the duration and severity of prostate cancer in C57BL/6 TRAMP x FVB mice does exist. Our interpretation of the grading scheme also could have led to scores that were lower than previously reported results. However, we believe that our results from the scoring scheme have given us tremendous insight into the effects of the treatment diets on PIN progression and prostate cancer development. 

Interestingly, there are some similarities between our findings and the outcomes from the finasteride and dutasteride clinical trials. The Pre-Finasteride group was designed to be similar to the participants of PCPT to determine whether finasteride could prevent prostate cancer development. Our findings were similar to PCPT; HG-PIN decreased and incidence of PD carcinoma increased. It is important to note that the effect of finasteride on progression to HG-PIN was weaker than dutasteride. It has been suggested that decreased prostate volume, biopsy density and/or prostate-specific antigen (PSA) performance may have contributed to the detection of more poorly differentiated prostate cancer in PCPT men [34-38], but our findings support that the increased incidence of high-grade prostate tumors seen in PCPT was an adverse effect of finasteride treatment.

The Pre-Dutasteride group was designed to be similar to the REDUCE trial, where the men who received dutasteride were at high risk of developing prostate cancer. Our findings were similar to the REDUCE trial in that the Pre-Dutasteride group had reduced HG-PIN incidence but increased incidence of PD carcinoma. The Post-Dutasteride group was designed to be similar to the REDEEM trial, in which assigned men with low-grade prostate cancer received dutasteride. Similar to the REDEEM trial, in the Post-Dutasteride group, HG-PIN incidence decreased without increasing the incidence of PD carcinoma, except in the lateral prostate.

Some caveats about our study should be considered, especially when interpreting what our findings might mean for the use of these drugs in men. First, the drugs were administered in diet, which differs from how most men take them. Second, the body weight–scaled human oral dose [39] is approximately 80 mg/day, which is much higher than the dutasteride (0.5 mg/day) and finasteride (5 mg/day) doses that most men take. In addition, the significant decrease in body weights in the dutasteride groups might mean that dietary-energy restriction might have contributed to the beneficial effects seen in these groups.

In conclusion, our results suggest that the timing of dutasteride treatment initiation may be critical to the risk of developing PD carcinoma. Our results may support therapeutic, but not preventive, use of dutasteride for this reason. Our results do not support the therapeutic or preventive use of finasteride. We plan to perform immunohistochemistry on prostates from these mice to elucidate why Post-Dutasteride treatment was effective and why we found a discordant response in the Pre-Dutasteride and both finasteride groups.
